# Handgrip strength and risk of cognitive outcomes: new prospective study and meta-analysis of 16 observational cohort studies

**DOI:** 10.1007/s11357-022-00514-6

**Published:** 2022-01-10

**Authors:** Setor K. Kunutsor, Nzechukwu M. Isiozor, Ari Voutilainen, Jari A. Laukkanen

**Affiliations:** 1 National Institute for Health Research Bristol Biomedical Research Centre, University Hospitals Bristol NHS Foundation Trust and University of Bristol, Bristol, UK; 2 Musculoskeletal Research Unit, Translational Health Sciences, Bristol Medical School, University of Bristol, Learning & Research Building (Level 1), Southmead Hospital, Bristol, BS10 5NB UK; 3 Diabetes Research Centre, University of Leicester, Leicester General Hospital, Gwendolen Road, Leicester, LE5 4WP UK; 4 Department of Medicine, Central Finland Health Care District Hospital District, Jyväskylä, Finland; 5 Institute of Clinical Medicine, Department of Medicine, University of Eastern Finland, Kuopio, Finland; 6 Institute of Public Health and Clinical Nutrition, University of Eastern Finland, Kuopio, Finland

**Keywords:** Handgrip strength, Cognitive impairment, Dementia, Alzheimer’s disease, Cohort study, Meta-analysis

## Abstract

Handgrip strength (HGS), a measure of muscular strength, might be a risk indicator for cognitive functioning, but the evidence is not consistent. Using a new prospective study and meta-analysis of published observational cohort studies, we aimed to evaluate the prospective associations of HGS with poor cognitive outcomes including cognitive impairment, dementia and Alzheimer’s disease (AD). Handgrip strength, measured using a Martin-Balloon-Vigorimeter, was assessed at baseline in a population-based sample of 852 men and women with good cognitive function in the Kuopio Ischemic Heart Disease cohort. Hazard ratios (HRs) with 95% confidence intervals (CIs) were estimated for cognitive outcomes. Relevant published studies were sought in MEDLINE, Embase and Web of Science from inception until October 2021 and pooled using random effects meta-analysis. During a median follow-up of 16.6 years, 229 dementia cases were recorded. Comparing extreme tertiles of HGS, the multivariable adjusted HR (95% CI) for dementia, AD and vascular dementia was 0.77 (0.55–1.07), 0.75 (0.52–1.10) and 0.49 (0.16–1.48), respectively. In a meta-analysis of 16 population-based prospective cohort studies (including the current study) comprising 180,920 participants, the pooled multivariable adjusted relative risks (95% CIs) comparing the top vs bottom thirds of HGS levels were as follows: 0.58 (0.52–0.65) for cognitive impairment; 0.37 (0.07–1.85) for cognitive decline; 0.73 (0.62–0.86) for dementia; 0.68 (0.53–0.87) for AD; and 0.48 (0.32–0.73) for vascular dementia. GRADE quality of evidence ranged from low to very low. Meta-analysis of aggregate prospective data suggests that HGS may be a risk indicator for poor cognitive outcomes such as cognitive impairment, dementia and AD. Systematic review registration: PROSPERO 2021: CRD42021237750.

**Supplementary Information:**

The online version contains supplementary material available at 10.1007/s11357-022-00514-6.

## Introduction

Cognitive impairment in older people is a transitional state that leads to dementia [17, 70]. Though cognitive decline is related to the ageing process, it goes beyond age-related cognitive changes, but it is characterised by less severe impairment compared to dementia [58]. In addition to marked deterioration of cognitive functions, dementia may be characterised by loss of independence and weakness. It represents a growing global public health burden and represents one of the major challenges of the century. Alzheimer’s disease (AD) is the most prevalent cause of dementia, with vascular dementia being the second most prevalent cause. Though ageing and the *APOE* gene represent the most well-known nonmodifiable risk factors for AD [20] and one-third of global AD cases globally are attributable to modifiable risk factors such as type 2 diabetes, hypertension, obesity, dyslipidemia, smoking, physical inactivity, smoking and depression [67], its pathogenesis is still not fully understood. The identification of individuals at elevated risk of dementia constitutes a difficult undertaking. There is therefore a need to identify easily measurable risk indicators that could aid in the early detection of poor cognitive functioning to implement intervention strategies.

The role of physical activity in improving health and reducing the risk of chronic disease outcomes such as vascular disease, diabetes and cancer is well known [42, 44, 48, 56]. The beneficial effects of physical activity on these outcomes have been reported to be modulated through its beneficial effects on risk factors such as body weight, glucose, blood pressure, lipid profiles and inflammation [41]. Physical activity has also been shown to promote brain plasticity and improve cognitive function [12]. Indeed, several studies have reported a decreased dementia risk with increased physical activity [23, 57]. In a comprehensive evidence-based review conducted by the American Academy of Neurology, it was concluded that exercise could be useful in slowing down the clinical progression from mild cognitive impairment (MCI) to dementia [68]. Physical fitness, which has cardiorespiratory fitness (CRF) and muscular fitness as its main components, is one of the strongest predictors of the future health status of an individual [2]. Muscular fitness also has muscular strength, muscular endurance and muscular power as its main components [2]. Cardiorespiratory fitness, an index of habitual aerobic physical activity [63], has also been consistently shown to be independently and inversely associated with the risk of several chronic disease outcomes including dementia [30, 47, 49, 83]. Handgrip strength (HGS), a proxy for muscular strength [4, 5] and a measure of physical fitness, has been recognised as a strong risk indicator for adverse health outcomes. Several observational cohort studies have shown HGS to be independently associated with reduced risk of several chronic disease outcomes as well as all-cause mortality [36, 46, 53, 54]. A number of epidemiological studies have evaluated the associations of HGS with the risk of poor cognitive outcomes such as cognitive impairment or decline, dementia and AD, but the results have been divergent [9, 25, 31, 59, 78, 81]. Whereas some studies have reported some associations [25, 31, 59], other studies have demonstrated no evidence of an association [9, 78, 81]. A number of these studies have also been based on cross-sectional and case–control designs, which lack temporality [29, 62]. There is uncertainty as to whether HGS could be a risk indicator for poor cognitive outcomes.

Given that HGS is easy to measure and convenient to use, it will be of immense clinical benefit if it can be used to identify individuals or patients at high risk of these outcomes. In this context, we aimed to re-evaluate the nature and magnitude of the associations of HGS with cognitive outcomes such as cognitive impairment, dementia and AD, using two approaches. First, we evaluated the associations using a population-based cohort study of men and women from eastern Finland followed up for over two decades. Second, we performed pooled analysis of available published prospective evidence on the association (including the new study), thereby offering the opportunity to assess the associations in a larger representative sample of participants.

## Materials and methods

### Prospective cohort methods

#### Study design and population

This study was reported in accordance with STROBE (STrengthening the Reporting of OBservational studies in Epidemiology) guidelines for reporting observational studies in epidemiology (Electronic Supplementary Material 1). Study participants for the cohort analysis utilised participants of the Kuopio Ischemic Heart Disease (KIHD) study, a population-based prospective cohort study designed in Kuopio, Finland, to investigate risk factors for vascular disease and other related outcomes [72]. Details of participant recruitment and flow have been reported previously (Electronic Supplementary Material 2) [45]. Briefly, the initial KIHD cohort comprised a representative sample of men aged 42–61 years recruited from Kuopio city and surrounding rural communities in eastern Finland. Re-examinations were conducted at 4 years, 11 years and 20 years after study entry. At the 11-year re-examinations, a randomly selected group of women aged 53–74 years was invited to join the initial cohort. Of this combined cohort, a subset of randomly selected participants had HGS assessments, who were utilised for this analysis [35, 45]. The current analysis employed 852 men and women with non-missing information on HGS, relevant covariates and cognitive outcomes.

### Ethics

The institutional review board of the University of Kuopio and Kuopio University Hospital, Kuopio, Finland (licence number 143/97), approved the research protocol. All study procedures were conducted according to the Declaration of Helsinki. All study participants provided written informed consent.

### Assessment of HGS and relevant covariates

A handheld dynamometer was used to assess the HGS of the dominant hand for each participant (in kPa; Martin-Balloon-Vigorimeter; Gebrüder Martin, Tuttlingen, Germany). This involved two measurements with the dynamometer calibrated at the beginning of each test and there was a 1-min resting gap between both HGS measurements. The mean of both values was used for analysis [36, 43, 52, 54]. Detailed description of physical measurements, assessment of lifestyle characteristics and prevalent diseases, blood sample collection and measurement of blood-based markers have been previously described [35, 39]. Self-administered questionnaires were used to assess lifestyle characteristics such as smoking and alcohol consumption, baseline diseases and use of medication. Before blood collection, participants fasted overnight and abstained from drinking alcohol for at least 3 days and smoking for at least 12 h. Circulating lipids were measured enzymatically (Boehringer Mannheim, Mannheim, Germany) from fresh serum samples after combined ultracentrifugation and precipitation [38]. Serum high sensitivity C-reactive protein (hsCRP) measurements were made with an immunometric assay (Immulite High Sensitivity C-Reactive Protein Assay; DPC, Los Angeles, CA, USA). Prevalent hypertension was defined as a physician diagnosis of hypertension, systolic blood pressure (SBP) ≥ 140 mmHg, diastolic blood pressure (DBP) ≥ 90 mmHg or use of antihypertensive medication. The energy expenditure of physical activity was assessed from a validated 12-month leisure-time physical activity questionnaire [51]. Body mass index (BMI) was calculated by dividing weight measured in kilogrammes by the square of height in metres.

### Ascertainment of cognitive outcomes

Dementia and AD cases that occurred from study entry through 2018 were included. There were no losses to follow-up as all KIHD study participants are under continuous annual monitoring (using personal identification codes) for incident outcomes including dementia cases as well as deaths [50]. Data on cognitive outcomes (dementia and AD) were ascertained from record linkage to the national computerised hospitalisation registry covering every specialised medical care hospitalisation and specialised medical care visit in Finland. Patients were initially screened using cognition tests including the Mini-Mental State Examination at baseline examination and once after the study entry. Screen-positives were then followed up for further testing. Those suspected of having dementia were examined by neurologists and had neuropsychological testing and magnetic resonance imaging of the brain [37, 40, 55]. The diagnoses of dementia cases were coded according to the International Classification of Diseases codes.

### Systematic review methods

#### Data sources and searches

We registered the systematic review in the PROSPERO prospective register of systematic reviews (CRD42021237750) and it was conducted in accordance with a predefined protocol and PRISMA and MOOSE guidelines [61, 79] (Electronic Supplementary Materials 3-4). MEDLINE and Embase were searched from inception to 06 October 2021 with no language restrictions placed on language. The computer-based searches used a combination of key MeSH terms or free text relating to the exposure (“handgrip strength”, “muscular strength”) and outcome (“cognitive impairment”, “cognitive decline”, “dementia”, “Alzheimer’s disease”). The detailed search strategy is reported in Electronic Supplementary Material 5. One author (S. K. K.) initially screened titles and abstracts of retrieved citations to assess their potential for inclusion. The screening was conducted using Rayyan, a free online bibliographic tool that helps expedite the initial screening of abstracts and titles using a process of semi-automation while incorporating a high level of usability [64]. This was then followed by full-text evaluation. To identify studies missed by the initial search, the reference lists of relevant studies and review articles were manually scanned and the Web of Science “cited reference search” was employed.

### Eligibility criteria

The protocol was pre-specified to include population-based observational cohort (retrospective or prospective, case cohort or nested case–control) studies conducted in general populations, had at least one year of follow-up and examined the relationship of HGS with the risk of incident cognitive outcomes such as cognitive impairment or decline, dementia or its subtypes in adult patients. The following studies were excluded: (i) case–control study designs and (ii) those in individuals with pre-existing cognitive impairment and dementia. To ensure consistency and enhance interpretation of the findings, we excluded studies that evaluated cognitive impairment as a continuous outcome, as these were reported on different scales.

### Data extraction and risk of bias assessment

Using a pre-tested standardised data collection form, one author (S. K. K.) initially extracted relevant data from eligible studies and a second author (N. M. I.) independently checked the data with that in original articles. Data were extracted on (i) study and design characteristics (first author and year of publication, country of origin, year of enrolment, study design, sample size and follow-up); demographic characteristics (age, sex); exposure (HGS tool and its assessment) and outcomes (type of event, its ascertainment and number, the most fully adjusted relative risks (RRs), hazard ratios (HRs) or odds ratios (ORs) with corresponding 95% confidence interval [CIs] and covariates adjusted for). For multiple articles involving the same cohort, study selection was limited to a single set of most comprehensive results to avoid double counting of study participants in the pooled analysis. The key factor used for selection was the most up-to-date and/or most comprehensively reported study.

The risk of bias within each observational study was assessed using the Cochrane Risk of Bias in Non-randomised Studies–of Interventions (ROBINS-I) tool [77]. This tool assesses risk of bias for confounding, participant selection, classification of interventions, deviations from intended interventions, missing data, outcome measurements and selective reporting. Risk is quantified in each domain as low risk, moderate risk, serious risk or critical risk, then an overall judgement of the risk of bias is provided for each study. We also used the Grading of Recommendations Assessment, Development and Evaluation (GRADE) approach to assess the quality of the body of evidence, based on study limitations, inconsistency of effect, imprecision, indirectness and publication bias [24].

### Statistical analyses

#### Prospective cohort analyses

Using descriptive analyses, baseline characteristics were summarised as means (standard deviation, SD) or medians (interquartile range, IQR) for continuous variables and percentages for categorical variables. Hazard ratios (HRs) with 95% confidence intervals (CIs) for dementia, AD and vascular dementia were estimated using Cox proportional hazard models. Handgrip strength was modelled as a continuous (per standard deviation (SD) increase) and categorical (tertiles) exposure variable. Hazard ratios were adjusted for in two progressive models: (i) age and sex and (ii) plus BMI, smoking status, history of type 2 diabetes mellitus (T2DM), history of hypertension, prevalent CHD, total cholesterol, high-density lipoprotein cholesterol (HDL-C), physical activity and hsCRP.

#### Meta-analysis

Relative risks with 95% CIs were used as the summary measures of association. To enable consistency and enhance pooling and interpretation of the results, reported study-specific risk estimates were converted to comparisons involving the top versus bottom tertiles of HGS values using standard statistical methods [11, 22] described in previous reports [33, 34, 36]. For comparisons that could not be transformed into extreme tertiles, the extreme groups (i.e. maximum versus minimal value of HGS) as provided by the reports were utilised for the analyses, as used in previous reports [36, 42, 44, 45]. This methodology is considered reliable as shown in a previous review, which showed that pooled estimates from transformed and untransformed data are qualitatively similar [10]. When the highest HGS was the referent, we converted the reported risk estimate into its reciprocal. The RRs were pooled using random effects models to account for the effect of between-study heterogeneity [14]. The outcomes (cognitive impairment, cognitive decline, dementia, AD and vascular dementia) were pooled separately as reported by the studies. For studies that reported only AD or vascular dementia, these were also classified as dementia in a separate analysis. Statistical heterogeneity between studies was quantified using standard chi-square tests and the *I*^2^ statistic [28]. Several study-level characteristics were used to investigate the sources of heterogeneity using stratified analysis and random effects meta-regression [80]. These included geographical location (Europe vs North America vs Asia), the average age at baseline (< 75 vs ≥ 75 years), the average duration of follow-up (< 10 vs ≥ 10 years, based on evidence suggesting that physical activity tends to decline approximately a decade before dementia diagnosis [71]), HGS assessment method (Jamar dynamometer vs other), number of events (< 250 vs ≥ 250), degree of adjustment (minimal adjustment ( +) vs adjustment for several established risk factors including comorbidities (+ +)) and overall risk of bias (moderate vs serious risk of bias). We assessed for small study effects using formal tests such as Begg’s funnel plots [3] and Egger’s regression symmetry test [16]. All statistical analyses were performed using Stata version MP 16 (Stata Corp, College Station, Texas).

## Results

### Prospective cohort

#### Baseline characteristics

The baseline characteristics of study participants are reported in Table 1. The overall mean (SD) age of study participants at baseline was 69 (3) years, with 47.4% being males. The mean (SD) baseline HGS was 76.2 (21.1) kPa. The prevalence of hypertension was 45.7% (389/852); of the 852 study participants, 277 (32.5%) had isolated systolic hypertension at study entry. During a median (IQR) follow-up of 16.6 (10.4–19.1) years, 229 dementia cases (annual rate 18.51/1,000 person-years at risk; 95% CI: 16.26 to 21.07) were recorded and these included 188 cases of AD and 22 cases of vascular dementia.

**Table 1:** Baseline participant characteristics

	Mean (SD), median (IQR) or *n* (%)
Handgrip strength (kPa)	76.2 (21.1)
***Questionnaire/prevalent conditions***	
Age at survey (years)	69 (3)
Males	404 (47.4)
History of type 2 diabetes	81 (9.5)
Current smokers	81 (9.5)
History of hypertension	389 (45.7)
History of CHD	306 (35.9)
***Physical measurements***	
BMI (kg/m^2^)	27.9 (4.3)
SBP (mmHg)	138 (18)
DBP (mmHg)	80 (9)
Energy expenditure of total LTPA (kcal/day)	378 (226–652)
***Blood-based markers***	
Total cholesterol (mmol/l)	5.44 (0.94)
HDL-C (mmol/l)	1.24 (0.32)
High-sensitivity CRP (mg/l)	1.59 (0.79–3.23)

*BMI* body mass index, *CHD* coronary heart disease, *CI* confidence interval, *CRP* C-reactive protein, *DBP* diastolic blood pressure, *HDL-C* high-density lipoprotein cholesterol, *IQR* interquartile range, *LTPA* leisure-time physical activity, *SD* standard deviation, *SBP* systolic blood pressure

### HGS and cognitive outcomes

The age- and sex-adjusted HR (95% CIs) for dementia per 1 SD increase in HGS was 0.90 (0.77–1.06) which was minimally attenuated to 0.94 (0.80–1.09) on further adjustment for BMI, smoking status, history of T2DM, history of hypertension, prevalent CHD, total cholesterol, HDL-C, physical activity and hsCRP. Alternatively, comparing the top versus bottom tertile of HGS values, the corresponding adjusted HRs (95% CIs) were 0.73 (0.53–1.02) and 0.77 (0.55–1.07), respectively (Table 2). Comparing the top versus bottom third of HGS, the fully adjusted HRs (95% CIs) for AD and vascular dementia were 0.75 (0.52–1.10) and 0.49 (0.16–1.48), respectively.

**Table 2:** Associations of handgrip strength with dementia, Alzheimer’s disease and vascular dementia in the KIHD prospective cohort

Handgrip strength (kPa)	Events/total	Model 1		Model 2	
		HR (95% CI)	*p-*value	HR (95% CI)	*p-*value
Dementia					
Per 1 SD increase	229/852	0.90 (0.77–1.06)	.20	0.94 (0.80–1.09)	.39
Tertile 1 (17–69)	84/286	1 [Reference]		1 [Reference]	
Tertile 2 (70–81)	79/283	0.86 (0.63–1.17)	.33	0.93 (0.68–1.27)	.64
Tertile 3 (82–442)	66/283	0.73 (0.53–1.02)	.07	0.77 (0.55–1.07)	.12
Alzheimer’s disease					
Per 1 SD increase	188/852	0.91 (0.76–1.08)	.28	0.94 (0.80–1.11)	.46
Tertile 1 (17–69)	68/286	1 [Reference]		1 [Reference]	
Tertile 2 (70–81)	69/283	0.93 (0.67–1.31)	.69	1.00 (0.71–1.41)	.98
Tertile 3 (82–442)	51/283	0.72 (0.50–1.05)	.09	0.75 (0.52–1.10)	.14
Vascular dementia					
Per 1 SD increase	22/852	0.58 (0.34–0.99)	.05	0.65 (0.38–1.12)	.12
Tertile 1 (17–69)	10/286	1 [Reference]		1 [Reference]	
Tertile 2 (70–81)	7/283	0.62 (0.24–1.64)	.34	0.73 (0.27–1.97)	.54
Tertile 3 (82–442)	5/283	0.42 (0.14–1.24)	.12	0.49 (0.16–1.48)	.20

*KIHD* Kuopio Ischemic Heart Study, *SD* standard deviation

Model 1: Adjusted for age and sex

Model 2: Model 1 plus body mass index, smoking status, history of type 2 diabetes mellitus, history of hypertension, prevalent coronary heart disease, total cholesterol, high-density lipoprotein cholesterol, physical activity and high sensitivity C-reactive protein

### Systematic review and meta-analysis

#### Study identification and selection

**Fig. 1:**
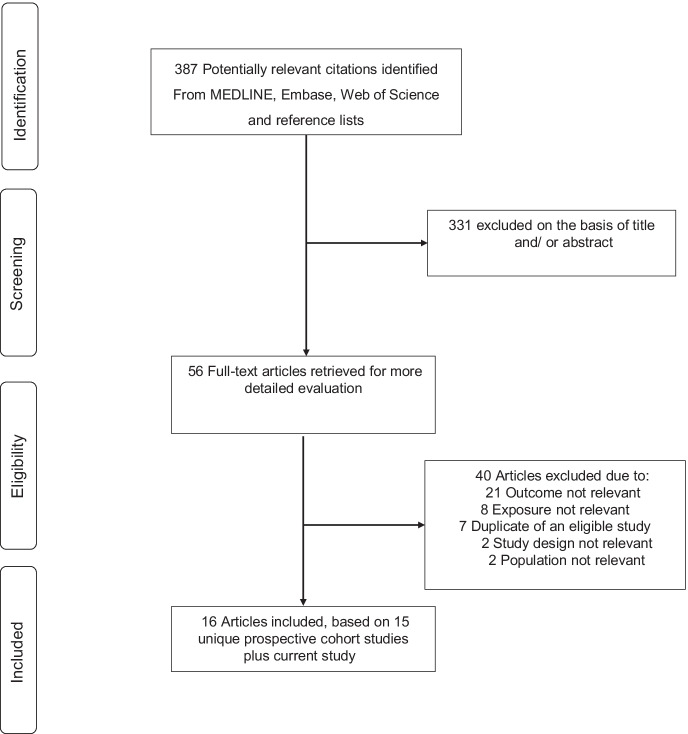
Study selection process

The study selection process is illustrated in Fig. 1. A total of 387 citations were retrieved from the search of the databases and manual reference screening of relevant articles. Following screening of titles and abstracts, 56 citations were selected for full text evaluation. Following detailed evaluation, 40 articles were excluded because: (i) outcome was not relevant (*n* = 21); (ii) exposure not relevant (*n* = 8); (iii) duplicate of an eligible study (*n* = 7); (iv) study design not relevant (*n* = 2); and (v) population not relevant (*n* = 2). We identified a total of 16 articles representing 15 unique prospective cohort studies [6–9, 15, 21, 25, 27, 31, 59, 66, 73, 75, 78, 81, 82]. Including the current study, the pooled analysis comprised 16 unique studies involving 180,920 participants.

#### Study characteristics and risk of bias

Table 3 summarises baseline characteristics of the eligible studies that evaluated the associations between HGS and cognitive outcomes in general populations. Publication years ranged from 2007 to 2020 and all the studies were based on prospective cohort designs. The average age at baseline ranged from approximately 58.0 to 85.0 years, with a weighted mean of 59.8 years. All studies enrolled both males and females. Five studies were based in Asia (Israel, Japan and South Korea); five in North America (USA); five in Europe (Finland, Germany, Italy and the UK); and one in Africa (Tanzania). Average duration of follow-up ranged from 2.0 to 25.0 years, with a weighted mean of 6.0 years. There was considerable variation in tools and methods of assessing HGS across studies; however, the Jamar handheld dynamometer was the most frequently used. Furthermore, most studies (*n* = 12) expressed HGS as kilogrammes. The most frequent cognitive outcomes evaluated by studies included cognitive impairment, cognitive decline, dementia and AD. Two studies reported on vascular dementia and one reported on the combined outcome of mild cognitive impairment and Alzheimer’s disease (MCI/AD). There was variation in the degree of covariate adjustment across studies, but most studies adjusted for conventional risk factors such as age, sex, education, physical activity, smoking, alcohol consumption, and prevalent comorbidities. Eight studies were at moderate risk of bias (i.e., at low or moderate risk of bias for all domains) and 8 studies were at serious risk of bias (i.e., were judged to be at serious risk of bias in at least one domain, but not at critical risk of bias in any domain) (Electronic Supplementary Material 6).

**Table 3:** Baseline characteristics of eligible prospective cohort studies (2007–2020)

Author, year of publication	Study	Country	Baseline year	Average age, years	Male %	Average follow-up, years	No. of participants	HGS assessment	Outcomes assessed	Covariates adjusted for
Buchman, 2007	Religious Orders Study	USA	1994–1996	74.4	30.7	5.7	877	Jamar handheld dynamometer	AD	Age, sex and education
Boyle, 2009	Rush Memory and Aging Project	USA	1997–2005	80.3	24.8	3.6	970	Jamar handheld dynamometer	AD	Age, sex and education
Yamada, 2009	RERF’s Adult Health Study	Japan	1992–1996	72.2	0.0	5.9	1637	NR	Dementia and AD	Age, age, education, BMI, smoking status, drinking status, menopausal age, and history of hypertension, DM or stroke
Boyle, 2010	Rush Memory and Aging Project	USA	1997–2005	79.0	24.0	3.6	761	Jamar handheld dynamometer	MCI	Age, sex and education
Sattler, 2011	ILSE	Germany	1993–1994	74.3	61.2	12.0	381	Martin-Vigorimeter	MCI/AD	Education, SES, gender and depressive symptoms
Gray, 2013	ACT	USA	1994–1996/2002–2004	76.8	39.9	6.5	2619	Handheld dynamometer	Dementia and AD	Age, sex, education, race, BMI, depressive symptoms, antidepressant use, self-reported health, hypertension, DM, MI, CHF, and smoking status and baseline Cognitive Abilities Screening Instrument score
Veronese, 2016	Progetto Veneto Anziani	Italy	1995–1997	72.2	40.4	4.4	1249	Jamar handheld dynamometer	Cognitive decline and impairment	Age, gender, BMI, preserved activities of daily living and instrumental activities of daily living, baseline scores in the Mini Mental State Examination and Geriatric Depression Scale; presence at the baseline of CVD, hypertension, osteoarthritis, fractures, chronic obstructive pulmonary disease, cancer; formal education; physical activity; smoking habits; monthly income
Camargo, 2016	Framingham Offspring	USA	1999–2005	62.0	45.0	11.0	2176	Jamar handheld dynamometer	Dementia and AD	Age, sex, DM, SBP, CVD, AF, smoking, WHR, total cholesterol, apolipoprotein E4 allele, total plasma homocysteine and physical activity
Stessman, 2017	Jerusalem Longitudinal Cohort Study	Israel	1990–1991	85.0	45.2	25.0	1187	5001 Grip-A handheld dynamometer	Cognitive impairment	Education, self-rated health, physical activity level, difficulty performing activities of daily living and DM
Sibbet, 2018	Lothian Birth Cohort 1921	UK	1999	79.0	42.6	9.7	488	Jamar handheld dynamometer	Dementia	FEV1, 6-m walk time, APOE ɛ4 carrier status, height, age, sex, history of hypertension, smoking status, age 11 IQ, history of cardiovascular or cerebrovascular disease and history of diabetes
Heward, 2018	IDEA	Tanzania	2014	76.2	43.1	2.0	305	Jamar handheld dynamometer	Cognitive decline	Age and sex
McGrath, 2019	HRS	USA	2006	≥ 50*		8.0	13,828	Smedley handheld dynamometer	Cognitive impairment	Age, sex, race and ethnicity, BMI, morbidity, Center for Epidemiologic Studies Depression score, smoking history, current smoking status, physical activity, social engagement, time (wave), educational achievement and self-rated health
Kim, 2019	KLoSA	South Korea	2006	63.4	45.5	8.0	5995	TANITA handheld dynamometer	Cognitive impairment	Time, baseline age, sex, education and quartiles of total household income as time-dependent covariates, time-varying smoking status, physical activity, alcohol consumption, obesity as time-dependent covariates, time-varying self-reported doctor diagnosis of co-morbidities, depressive symptoms and engagement in social activities as time-dependent covariates
Doi, 2019	NCG and GSGS	Japan	2011–2012	72.0	48.0	3.6	4086	Smedley handheld dynamometer	Dementia	Age, sex, educational history, BMI, diseases, number of medications, falls, current smoking, alcohol consumption, cognitive function and physical inactivity
Hatabe, 2020	Hisayama study	Japan	1988	68.0	42.6	14.6	1055	Smedley handheld dynamometer	Dementia, AD and VD	Age, sex, education level, SBP, use of antihypertensive agents, DM, total cholesterol, body mass index, electrocardiogram abnormalities, smoking habit, alcohol intake and regular exercise
Petermann-Rocha, 2020	UK Biobank	UK	2006–2010	58.0	46.0	5.4	143,215	Jamar handheld dynamometer	Dementia	Age, sex, deprivation, ethnicity, education, leisure or social activities, frequency of friend and family visits, smoking, sleep duration, total discretionary sedentary time, alcohol intake, and consumption of red meat, processed meat, and fruit and vegetables, BMI, morbidity count, blood pressure, total cholesterol, glycated haemoglobin, reaction time at baseline and other components of sarcopenia
Current study	KIHD	Finland	1998–2001	69.0	47.4	16.6	852	Martin-Vigorimeter	Dementia, AD and VD	Age, sex, BMI, smoking status, history of type 2 DM, prevalent CHD, total cholesterol, HDL-C, physical activity and hsCRP

^*^Age range provided

*AD* Alzheimer’s disease, *AF* atrial fibrillation, *BMI* body mass index, *CHD* coronary heart disease, *CHF* congestive heart failure, *CVD* cardiovascular disease, *DM* diabetes mellitus, *FEV1* forced expiratory volume, *HDL-C* high-density lipoprotein cholesterol, *HGS* handgrip strength, *MCI* mild cognitive impairment, *MI* myocardial infarction, *NR* not reported, *SES* socioeconomic status, *SBP* systolic blood pressure, *VD* vascular dementia, *WHR* waist-to-hip ratio

Study abbreviations: ACT, Adult Changes in Thought; HRS, Health and Retirement Study; IDEA, Intervention for Dementia in Elderly Africans; ILSE, Interdisciplinary Longitudinal Study on Adult Development and Aging;

### HGS and cognitive outcomes

**Fig. 2:**
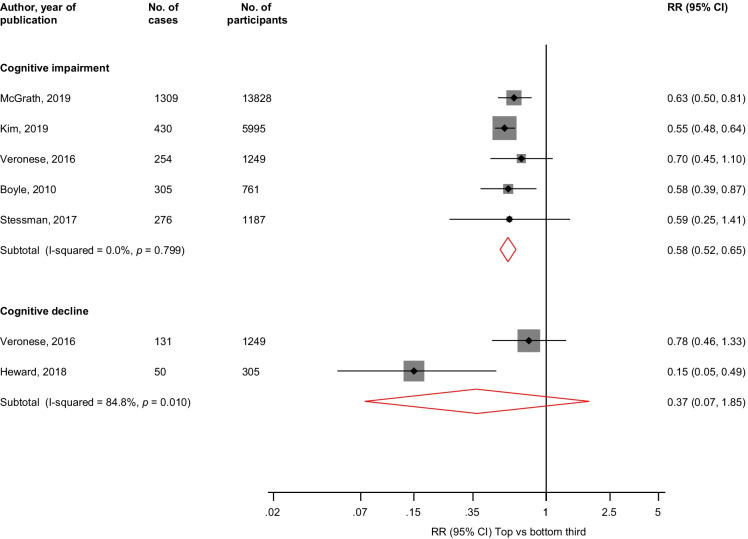
Association between handgrip strength and risk of cognitive impairment and decline. The summary estimates presented were calculated using random effects models; relative risks are reported comparing extreme tertiles of handgrip strength; CI, confidence interval (bars); HGS, handgrip strength; RR, relative risk

In pooled analysis of 5 studies, the multivariable adjusted RR (95% CI) of cognitive impairment (2574 cases) comparing the top versus bottom third of HGS values was 0.58 (0.52–0.65; *I*^2^ = 0%; 95% CI: 0, 79%; *p* < 0.001) (Fig. 2). The corresponding RR (95% CI) for cognitive decline (181 cases) in pooled analysis of 2 studies was 0.37 (0.07–1.85).

**Fig. 3:**
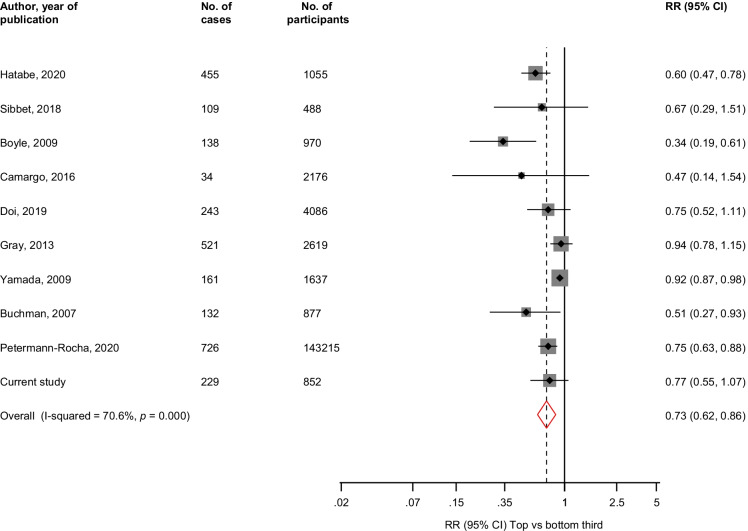
Association between handgrip strength and risk of dementia. The summary estimates presented were calculated using random effects models; relative risks are reported comparing extreme tertiles of handgrip strength; CI, confidence interval (bars); HGS, handgrip strength; RR, relative risk

**Fig. 4:**
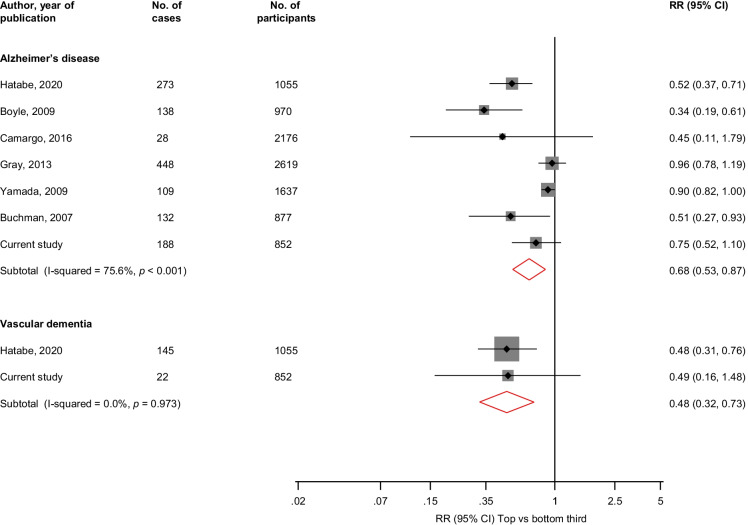
Associations between handgrip strength and risk of Alzheimer’s disease and vascular dementia. The summary estimates presented were calculated using random effects models; relative risks are reported comparing extreme tertiles of handgrip strength; CI, confidence interval (bars); HGS, handgrip strength; RR, relative risk

The pooled multivariable adjusted RR (95% CI) comparing the top vs bottom thirds of HGS levels was 0.73 (0.62–0.86; *I*^2^ = 71%; 95% CI: 44, 85%; *p* < 0.001) for dementia (10 studies, 2748 cases) (Fig. 3). The corresponding RRs (95% CIs) were 0.68 (0.53–0.87; *I*^2^ = 76%; 95% CI: 48, 88%; *p* < 0.001) for AD (7 studies, 1316 cases) and 0.48 (0.32–0.73) for vascular dementia (2 studies, 167 cases) (Fig. 4). A single study reported that HGS was not associated with the combined outcome of MCI/AD (126 cases) [73].

### Subgroup analysis and assessment of publication bias

The inverse association between HGS and dementia risk was consistent across several subgroups, except for significant evidence of effect modification by degree of adjustment (*p*-value for meta-regression = 0.005); the associations were stronger for minimally adjusted studies than those adjusted for several established risk factors plus comorbidity studies (Electronic Supplementary Material 7). For the outcome of dementia (*n* = 10 studies), Egger’s test for publication bias was significant (*p* = 0.008), consistent with observed funnel plot asymmetry (Electronic Supplementary Material 8), suggesting that studies with less striking results were less likely to have been reported. Despite the concern that small studies with null results often tend not to be published, we found no clear evidence of such selective reporting when studies were grouped by size in meta-regression analysis (Electronic Supplementary Material 7). Duval and Tweedie’s trim-and-fill method, which was used to adjust for publication bias, imputed five additional studies (Electronic Supplementary Material 9). The pooled RR (95% CI) following adjustment for publication bias was 0.84 (0.68–1.04).

### GRADE summary of findings

GRADE ratings for all cognitive outcomes were assessed and are reported in Electronic Supplementary Material 10. GRADE quality of the evidence ranged from low to very low.

## Discussion

### Key findings

Handgrip strength is a powerful marker of ageing [60]. Though it is well established that ageing results in a decline in physical and cognitive abilities, the evidence is uncertain as to whether HGS is a risk indicator for poor cognitive functioning. Evidence on the associations of HGS with cognitive outcomes have so far been conflicting. Our analysis of a new population-based prospective study of 852 older Finnish men and women with good cognitive function at baseline showed no significant evidence of associations between HGS and cognitive outcomes, which are likely due to the low event rates. On pooling evidence from 15 general population-based prospective cohort studies plus the current study, there was evidence of associations between increased levels of HGS and lower risk of cognitive impairment, dementia, AD and vascular dementia. There was no evidence of an association with cognitive decline, which was based on pooled analysis of only two studies. The association between HGS and dementia remained consistent across several relevant subgroups, except for evidence of effect modification by degree of adjustment; as expected, the association was stronger in studies that adjusted for fewer covariates. The quality of the evidence ranged from low to very low.

### Comparison with previous work

In a narrative scoping review of 15 prospective studies cohort studies to determine the relationship between HGS and cognitive decline over time, Fritz and colleagues [18] concluded that the reviewed studies provided strong support for measuring HGS to monitor the progression of patients with cognitive decline. There was a call for the conduct of a systematic review to explore the longitudinal relationships between handgrip strength and cognitive performance. Kobayashi-Cuya and colleagues [32] in their systematic review of 22 observational cross-sectional and longitudinal studies reported that HGS was associated with cognitive performance; however, they acknowledged lack of clarity regarding which variable affected the other in the long-term. In another systematic review of 6 observational cross-sectional and longitudinal studies, the main observations were that although cognitive function and HGS declined on average in later life, their declines were not necessarily associated [85]. In a narrative review of cross-sectional and longitudinal studies evaluating HGS and cognitive functioning, Shaughnessy and colleagues [74] concluded that a relationship existed. Previous reviews on the association between HGS and poor cognitive functioning have relied on narrative synthesis of the existing data, inclusion of cross-sectional study designs and evaluation of few selected outcome measures such as cognitive function or decline. Our inclusion of only prospective cohort designs enabled evaluation of the temporal associations. We were able to harmonise and pool the data, which enabled quantification of the nature and magnitude of the associations. We also included a wide range of adverse cognitive outcomes as reported by the included studies.

### Mechanistic pathways underlying findings

Several potential mechanisms of action may underlie the protective effect of HGS on the risk of cognitive impairment or dementia. Inflammation and oxidative stress are implicated in the pathogenesis of dementia [19, 84]; there is evidence suggesting that the loss of skeletal muscle is associated with high levels of inflammatory markers such as interleukin-6 and CRP [1]. The skeletal muscle is recognised as a secretory organ; the cytokines and peptides (classified as myokines) produced by the skeletal muscle are dependent on contraction [65]. Hence, physical inactivity may lead to altered myokine response, which could be the underlying mechanism between sedentary behaviour and chronic diseases such as dementia [65]. Myokines such as brain-derived neurotrophic factor and insulin-like growth factor-1 have been reported to play a role in learning and neural plasticity [65]. Handgrip strength is an indicator of frailty [4], which is usually associated with fatigue, reduced muscle mass and high susceptibility to chronic diseases such as cardiovascular disease (CVD) and dementia. Muscle weakness and loss is associated with vitamin D deficiency, which plays a role in the development of several chronic diseases, including dementia [13]. Though the eligible studies did not include participants with pre-existing cognitive dysfunction during study entry, it is still possible that these observational findings could be to reverse causality, i.e. dementia causing lower HGS. It is known that physical activity tends to decline in the early phases of dementia before clinical diagnosis [71], physical inactivity over time leads to loss of muscle mass and strength. Indeed, in a study to assess the bi-directional relationship between HGS and cognitive impairment, Kim and colleagues [31] collected repeated measures of HGS and cognitive function over a period of 8 years and showed a significant bi-directional relationship between muscular strength and cognitive function. Though it is likely that the null associations between HGS and cognitive outcomes in our primary cohort could be attributed to the low event rate, other likely reasons for the heterogeneous results in prior studies and that of our primary cohort study could be due to differences in study design features and population characteristics such as (i the follow-up duration; (ii age, sex, race or genetic background of participants; and (iii assessment of HGS and case definition of cognitive outcomes or a combination of all of these. Studies may have been affected by reverse causation bias, as evidence suggests physical activity declines in the early phases of dementia before clinical diagnosis [71]. Consistent with this observation, significant findings were demonstrated predominantly in studies which short-term follow-up durations [6, 8, 66, 82] and null associations in long-term follow-up studies [9, 73] including our primary cohort. Furthermore, due to the phenomenon of regression dilution bias, longer-term follow-up studies are commonly characterised by null findings. Studies with elderly populations are more likely to be affected by reverse causation bias; many of the included studies that demonstrated significant associations between HGS and cognitive outcomes were based in older participants [6, 7, 27]. Whether sex could be an effect modifier of the association between HGS and dementia risk is uncertain, as no study has specifically evaluated this. One study assessed the associations of HGS with both cognitive decline and impairment in men and women separately and found no significant evidence of associations in both genders [81].

### Implications of findings

Findings of associations between elevated HGS and the decreased risk of poor cognitive outcomes may have important clinical implications, especially in the area of developing preventive strategies for cognitive dysfunction. Handgrip strength, a quick, easy-to-use and low-cost tool, could be used as a potential risk assessment tool to identify individuals at risk of cognitive dysfunction, which could trigger the adoption of targeted lifestyle changes. We have recently shown that information on HGS augments the risk prediction of T2DM and CVD mortality beyond that of traditional risk factors in the general population [46, 53]. Formal risk prediction evaluations using large-scale prospective studies are urgently needed to demonstrate if HGS can be potentially used as a risk assessment tool for poor cognitive outcomes in general population settings. Apart from aerobic physical activity, activities such as resistance training which can be used to increase muscle strength should be widely encouraged. Though the evidence on the association between physical activity and the risk of dementia is still inconclusive [41], there is overwhelming evidence on the beneficial effects of physical activity on overall health.

### Strengths and limitations

Other strengths in addition to those listed above include (i) use of a new population study which comprised a well-characterised cohort of men and women who were nationally representative in the age group considered; (ii) HGS was assessed using the Martin-Vigorimeter, which is known for its high reliability and accuracy, in assessing grip strength especially in the geriatric population [76]; (iii) the long and complete follow-up of the cohort; (iv) the novel approach of conducting a pooled analysis of previous cohort studies including the current study, to put the findings into wider context; and (v) our systematic review and meta-analysis involved the transformation of reported risk estimates to consistent comparisons using standard methods (which enhanced the pooling process for easy interpretation); there was exploration for small study effects and for sources of heterogeneity using clinically relevant characteristics, and the assessment of the risk of bias and the quality of the evidence using well-established tools. We acknowledge some very important limitations which were inherent to the studies as well as the use of aggregate data, some of which could bias the study findings. We were unable to transform some of the risk estimates to extreme tertiles; hence, comparisons could only be made between the maximum versus minimum value of HGS. Nevertheless, we have shown in a previous review that pooled results from untransformed data of extreme categories are not very different from those based on transformed data [10]. The HGS assessment methods varied across studies; however, most studies used the Jamar handheld dynamometer. Roberts and colleagues in their comprehensive review of the measurement of grip strength in clinical and epidemiological studies demonstrated considerable variation in methods of assessing HGS and acknowledged the difficulties in making comparisons between studies [69]. Our subgroup analysis showed no evidence of effect modification on the association by the type of HGS assessment method. Given that the percentage of people with dementia increases dramatically with age (3% of people age 65–74, 17% of people age 75–84 and 32% of people age 85 or older [26]), the potentially long but unclear latency period for dementia and the relatively lower average age of participants and shorter follow-up durations of most of the studies included in the meta-analysis, there was a potential for under-reporting of dementia events, yielding lower incidence estimates of the outcomes. Sex-specific associations could not be evaluated as the included studies did not provide these results. We could not evaluate the impact of a uniform approach to statistical adjustment such as the model employed in our primary analysis, because the degree of adjustment varied across studies. We could not evaluate the actual dose–response relationship of the association because of the heterogeneous nature of the HGS data. Finally, our associations could have been underestimated due to the potential for regression dilution bias, as HGS measurements used by studies were mostly baseline values and a number of studies had long follow-up durations (> 10 years). Despite these limitations, the findings suggest a potential role of utilising HGS measurements as an easily available clinical measure in the prevention of cognitive dysfunction and also warrant further investigation.

## Conclusion

Handgrip strength was only modestly associated with risk of cognitive outcomes in the primary cohort analysis, which may be driven by the low event rate. Meta-analysis of aggregate prospective data suggests that HGS may be a risk indicator for poor cognitive outcomes such as cognitive impairment, dementia and AD.

## Supplementary Information

Below is the link to the electronic supplementary material.

Supplementary file1 (DOCX 432 KB)

## Declarations

### Disclaimer

The funder had no role in study design, data collection and analysis, decision to publish or preparation of the manuscript.
