# Supplementary material for: Handgrip strength and risk of cognitive outcomes: new prospective study and meta-analysis of 16 observational cohort studies
Source: GeroScience. 2022 Jan 10;44(4):2007–24. doi: 10.1007/s11357-022-00514-6 (PMC9616970; doi:10.1007/s11357-022-00514-6)
Supplement: Supplementary file 1 — Supplementary file1 (DOCX 432 KB) [file 11357_2022_514_MOESM1_ESM.docx]

**ELECTRONIC SUPPLEMENTARY MATERIAL**

**Handgrip strength and risk of cognitive outcomes: new prospective study and meta-analysis of 16 observational cohort studies**

Setor K. Kunutsor^1,2,3,4^, Nzechukwu M. Isiozor^5^, Ari Voutilainen^5^, Jari A. Laukkanen^4,5,6^

^1^National Institute for Health Research Bristol Biomedical Research Centre, University Hospitals Bristol NHS Foundation Trust and University of Bristol, Bristol, UK

^2^Musculoskeletal Research Unit, Translational Health Sciences, Bristol Medical School, University of Bristol, Learning & Research Building (Level 1), Southmead Hospital, Bristol, BS10 5NB, UK

^4^Diabetes Research Centre, University of Leicester, Leicester General Hospital, Gwendolen Road, Leicester, LE5 4WP, UK

^4^Central Finland Health Care District Hospital District, Department of Medicine, Jyväskylä, Finland

District, Jyväskylä, Finland

^5^Institute of Public Health and Clinical Nutrition, University of Eastern Finland, Kuopio, Finland

^6^Institute of Clinical Medicine, Department of Medicine, University of Eastern Finland, Kuopio, Finland

**Correspondence**

Setor K. Kunutsor, Musculoskeletal Research Unit, Translational Health Sciences, Bristol Medical School, University of Bristol, Learning & Research Building (Level 1), Southmead Hospital, Bristol, BS10 5NB, UK; Phone: +44-7539589186; Fax: +44-1174147924; Email address: [skk31@cantab.net](mailto:skk31@cantab.net); ORCID: 0000-0002-2625-0273

| **Electronic Supplementary Material 1** | STROBE Statement |
| --- | --- |
| **Electronic Supplementary Material 2** | Participant flow in prospective cohort |
| **Electronic Supplementary Material 3** | PRISMA checklist |
| **Electronic Supplementary Material 4** | MOOSE checklist |
| **Electronic Supplementary Material 5** | Literature search strategy |
| **Electronic Supplementary Material 6** | Risk of bias assessments |
| **Electronic Supplementary Material 7** | Association between handgrip strength and risk of dementia, grouped by study-level characteristics |
| **Electronic Supplementary Material 8** | Assessment of small study effects by funnel plot |
| **Electronic Supplementary Material 9** | Trim-and-fill analysis |
| **Electronic Supplementary Material 10** | GRADE summary of findings |

**Electronic Supplementary Material 1** STROBE Statement

| **Section/Topic** | Item # | Recommendation | Reported on page # |
| --- | --- | --- | --- |
| **Title and abstract** | 1 | (*a*) Indicate the study’s design with a commonly used term in the title or the abstract | Page 1 |
|  |  | (*b*) Provide in the abstract an informative and balanced summary of what was done and what was found | Page 2 |
| Introduction | | |  |
| Background/rationale | 2 | Explain the scientific background and rationale for the investigation being reported | Page 3-4 |
| Objectives | 3 | State specific objectives, including any pre-specified hypotheses | Page 3-4 |
| Methods | | |  |
| Study design | 4 | Present key elements of study design early in the paper | Study design and population |
| Setting | 5 | Describe the setting, locations, and relevant dates, including periods of recruitment, exposure, follow-up, and data collection | Study design and population |
| Participants | 6 | (*a*) Give the eligibility criteria, and the sources and methods of selection of participants. Describe methods of follow-up | Study design and population |
|  |  | (*b*) For matched studies, give matching criteria and number of exposed and unexposed | Not applicable |
| Variables | 7 | Clearly define all outcomes, exposures, predictors, potential confounders, and effect modifiers. Give diagnostic criteria, if applicable | Assessment of handgrip strength and relevant covariates |
| Data sources/ measurement | 8* | For each variable of interest, give sources of data and details of methods of assessment (measurement). Describe comparability of assessment methods if there is more than one group | Assessment of handgrip strength and relevant covariates |
| Bias | 9 | Describe any efforts to address potential sources of bias | Statistical analyses |
| Study size | 10 | Explain how the study size was arrived at | Statistical analyses |
| Quantitative variables | 11 | Explain how quantitative variables were handled in the analyses. If applicable, describe which groupings were chosen and why | Statistical analyses |
| Statistical methods | 12 | (*a*) Describe all statistical methods, including those used to control for confounding | Statistical analyses |
|  |  | (*b*) Describe any methods used to examine subgroups and interactions | Statistical analyses |
|  |  | (*c*) Explain how missing data were addressed | Not applicable |
|  |  | (*d*) If applicable, explain how loss to follow-up was addressed | Not applicable |
|  |  | (*e*) Describe any sensitivity analyses | Statistical analyses |
| Results | | |  |
| Participants | 13* | (a) Report numbers of individuals at each stage of study—eg numbers potentially eligible, examined for eligibility, confirmed eligible, included in the study, completing follow-up, and analysed | Study design and population |
|  |  | (b) Give reasons for non-participation at each stage | Study design and population |
|  |  | (c) Consider use of a flow diagram | Study design and population |
| Descriptive data | 14* | (a) Give characteristics of study participants (eg demographic, clinical, social) and information on exposures and potential confounders | Results; Tables 1 |
|  |  | (b) Indicate number of participants with missing data for each variable of interest |  |
|  |  | (c) Summarise follow-up time (eg, average and total amount) | Results |
| Outcome data | 15* | Report numbers of outcome events or summary measures over time | Results |
| Main results | 16 | (*a*) Give unadjusted estimates and, if applicable, confounder-adjusted estimates and their precision (eg, 95% confidence interval). Make clear which confounders were adjusted for and why they were included | Results; Table 2 |
|  |  | (*b*) Report category boundaries when continuous variables were categorized | Results; Table 2 |
|  |  | (*c*) If relevant, consider translating estimates of relative risk into absolute risk for a meaningful time period |  |
| Other analyses | 17 | Report other analyses done—eg analyses of subgroups and interactions, and sensitivity analyses | Results |
| Discussion |  |  |  |
| Key results | 18 | Summarise key results with reference to study objectives | Discussion - Summary of main findings |
| **Limitations** |  |  |  |
| Interpretation | 20 | Give a cautious overall interpretation of results considering objectives, limitations, multiplicity of analyses, results from similar studies, and other relevant evidence | Discussion |
| Generalisability | 21 | Discuss the generalisability (external validity) of the study results | Discussion |
| Other information |  |  |  |
| Funding | 22 | Give the source of funding and the role of the funders for the present study and, if applicable, for the original study on which the present article is based | Provided |

**Electronic Supplementary Material 2** Participant flow in prospective cohort

**Electronic Supplementary Material 3** PRISMA checklist

| **Section/topic** | **Item No** | **Checklist item** | **Reported on page No** |
| --- | --- | --- | --- |
| **Title** | | | |
| Title | 1 | Identify the report as a systematic review, meta-analysis, or both | 1 |
| **Abstract** | | | |
| Structured summary | 2 | Provide a structured summary including, as applicable, background, objectives, data sources, study eligibility criteria, participants, interventions, study appraisal and synthesis methods, results, limitations, conclusions and implications of key findings, systematic review registration number | 2 |
| **Introduction** | | | |
| Rationale | 3 | Describe the rationale for the review in the context of what is already known | 4 |
| Objectives | 4 | Provide an explicit statement of questions being addressed with reference to participants, interventions, comparisons, outcomes, and study design (PICOS) | 4 |
| **Methods** | | | |
| Protocol and registration | 5 | Indicate if a review protocol exists, if and where it can be accessed (such as web address), and, if available, provide registration information including registration number | Methods |
| Eligibility criteria | 6 | Specify study characteristics (such as PICOS, length of follow-up) and report characteristics (such as years considered, language, publication status) used as criteria for eligibility, giving rationale | Methods |
| Information sources | 7 | Describe all information sources (such as databases with dates of coverage, contact with study authors to identify additional studies) in the search and date last searched | Methods |
| Search | 8 | Present full electronic search strategy for at least one database, including any limits used, such that it could be repeated | ESM 5 |
| Study selection | 9 | State the process for selecting studies (that is, screening, eligibility, included in systematic review, and, if applicable, included in the meta-analysis) | Methods |
| Data collection process | 10 | Describe method of data extraction from reports (such as piloted forms, independently, in duplicate) and any processes for obtaining and confirming data from investigators | Methods |
| Data items | 11 | List and define all variables for which data were sought (such as PICOS, funding sources) and any assumptions and simplifications made | Methods |
| Risk of bias in individual studies | 12 | Describe methods used for assessing risk of bias of individual studies (including specification of whether this was done at the study or outcome level), and how this information is to be used in any data synthesis | Methods |
| Summary measures | 13 | State the principal summary measures (such as risk ratio, difference in means). | Methods |
| Synthesis of results | 14 | Describe the methods of handling data and combining results of studies, if done, including measures of consistency (such as I^2^ statistic) for each meta-analysis | Methods |
| Risk of bias across studies | 15 | Specify any assessment of risk of bias that may affect the cumulative evidence (such as publication bias, selective reporting within studies) | Methods |
| Additional analyses | 16 | Describe methods of additional analyses (such as sensitivity or subgroup analyses, meta-regression), if done, indicating which were pre-specified | Methods |
| **Results** | | | |
| Study selection | 17 | Give numbers of studies screened, assessed for eligibility, and included in the review, with reasons for exclusions at each stage, ideally with a flow diagram | Figure 1 |
| Study characteristics | 18 | For each study, present characteristics for which data were extracted (such as study size, PICOS, follow-up period) and provide the citations | Table 3 |
| Risk of bias within studies | 19 | Present data on risk of bias of each study and, if available, any outcome-level assessment (see item 12). | Table 3 |
| Results of individual studies | 20 | For all outcomes considered (benefits or harms), present for each study (a) simple summary data for each intervention group and (b) effect estimates and confidence intervals, ideally with a forest plot | Figures 2-3 |
| Synthesis of results | 21 | Present results of each meta-analysis done, including confidence intervals and measures of consistency | Results and Figures 2-3 |
| Risk of bias across studies | 22 | Present results of any assessment of risk of bias across studies (see item 15) | ESM 6 |
| Additional analysis | 23 | Give results of additional analyses, if done (such as sensitivity or subgroup analyses, meta-regression) (see item 16) | ESM 7-9 |
| **Discussion** | | | |
| Summary of evidence | 24 | Summarise the main findings including the strength of evidence for each main outcome; consider their relevance to key groups (such as health care providers, users, and policy makers) | Discussion |
| Limitations | 25 | Discuss limitations at study and outcome level (such as risk of bias), and at review level (such as incomplete retrieval of identified research, reporting bias) | Discussion |
| Conclusions | 26 | Provide a general interpretation of the results in the context of other evidence, and implications for future research | Discussion |
| **Funding** | | | |
| Funding | 27 | Describe sources of funding for the systematic review and other support (such as supply of data) and role of funders for the systematic review | Provided |

**Electronic Supplementary Material 4** MOOSE checklist

| **Criteria** | | **Brief description of how the criteria were handled in the review** |
| --- | --- | --- |
| **Reporting of background** | |  |
| √ | Problem definition | There is diverging evidence on the associations between handgrip strength and cognitive outcomes |
| √ | Hypothesis statement | Elevated handgrip strength is associated with lower risk of poor cognitive outcomes |
| √ | Description of study outcomes | Cognitive impairment or decline, dementia, Alzheimer’s disease, vascular dementia |
| √ | Type of exposure | Handgrip strength |
| √ | Type of study designs used | Prospective (cohort, case-cohort or “nested case control”) population-based studies |
| √ | Study population | Approximately general populations |
| **Reporting of search strategy should include** | |  |
| √ | Qualifications of searchers | Setor Kunutsor, MD PhD; Nzechukwu Isiozor, MD |
| √ | Search strategy, including time period included in the synthesis and keywords | Time period: from inception of MEDLINE and EMBASE to 06 October 2021  **Search strategy:**  In Electronic Supplementary Material 5 |
| √ | Databases and registries searched | MEDLINE, Embase and Web of Science citation search |
| √ | Search software used, name and version, including special features | Ovid was used to search MEDLINE and Embase  Endnote used to manage references |
| √ | Use of hand searching | We searched bibliographies of retrieved papers |
| √ | List of citations located and those excluded, including justifications | Details of the literature search process are outlined in the flow chart. The citation list for excluded studies is available upon request. |
| √ | Method of addressing articles published in languages other than English | We placed no restrictions on language |
| √ | Method of handling abstracts and unpublished studies | None found |
| √ | Description of any contact with authors | Not applicable |
| **Reporting of methods should include** | |  |
| √ | Description of relevance or appropriateness of studies assembled for assessing the hypothesis to be tested | Detailed inclusion and exclusion criteria are described in the Methods section. |
| √ | Rationale for the selection and coding of data | Data extracted from each of the studies were relevant to the population characteristics, study design, exposure, outcome, and possible effect modifiers of the association. |
| √ | Assessment of confounding | Fully adjusted risk estimates were used. |
| √ | Assessment of study quality, including blinding of quality assessors; stratification or regression on possible predictors of study results | The risk of bias within each observational study was assessed using the Cochrane Risk of Bias in Non-randomised Studies – of Interventions (ROBINS-I) tool |
| √ | Assessment of heterogeneity | Heterogeneity of the studies was explored with I^2^ statistic that provides the relative amount of variance of the summary effect due to the between-study heterogeneity. |
| √ | Description of statistical methods in sufficient detail to be replicated | Description of methods are detailed in the methods. We performed random effects meta-analysis with Stata MP 16 |
| √ | Provision of appropriate tables and graphics | Table 3 |
| **Reporting of results should include** | |  |
| √ | Graph summarizing individual study estimates and overall estimate | Figures 2-4 |
| √ | Table giving descriptive information for each study included | Table 3 |
| √ | Results of sensitivity testing | Results; ESM 7-9 |
| √ | Indication of statistical uncertainty of findings | 95% confidence intervals were presented with all summary estimates, I^2^ values and results of sensitivity analyses |
| **Reporting of discussion should include** | |  |
| √ | Quantitative assessment of bias | Discussed |
| √ | Justification for exclusion | All studies were excluded based on the pre-defined inclusion criteria in methods section. |
| √ | Assessment of quality of included studies | Brief discussion included in ‘Methods’ section |
| **Reporting of conclusions should include** | |  |
| √ | Consideration of alternative explanations for observed results | Discussed in the context of the results. |
| √ | Generalization of the conclusions | Discussed in the context of the results. |
| √ | Guidelines for future research | Further large-scale studies are needed |
| √ | Disclosure of funding source | None |

**Electronic Supplementary material 5** Literature search strategy

| Relevant studies, published before 06 October 2021 (date last searched), were identified through electronic searches not limited to the English language using MEDLINE and Embase databases. Electronic searches were supplemented by scanning reference lists of articles identified for all relevant studies (including review articles) and by hand searching of relevant journals. The computer-based searches combined search terms related to handgrip strength and fracture without language restriction.  1 Hand Strength/ (15676)  2 grip strength.mp. (12027)  3 Muscle Strength/ or musc* strength.mp. (38762)  4 cognitive impairment.mp. or exp Cognitive Dysfunction/ (68800)  5 cognitive function.mp. (37732)  6 exp Dementia/ (171811)  7 exp Alzheimer Disease/ (97370)  8 cohort studies/ or longitudinal studies/ or follow-up studies/ or prospective studies/ or retrospective studies/ or cohort.ti,ab. or longitudinal.ti,ab. or prospective.ti,ab. or retrospective.ti,ab. (2739587)  9 1 or 2 or 3 (58275)  10 4 or 5 or 6 or 7 (245541)  11 8 and 9 and 10 (320)  12 limit 11 to humans (276) |
| --- |

**Electronic Supplementary material 6** Risk of bias assessments

**Electronic Supplementary material 7** Association between handgrip strength and risk of dementia, grouped by study-level characteristics

CI, confidence interval (bars); RR, relative risk; *, *p*-value for meta-regression; +, minimal adjustment; ++, adjustment for several established risk factors including comorbidities

**Electronic Supplementary material 8** Assessment of small study effects by funnel plot

**Electronic Supplementary material 9** Trim-and-fill analysis

**Electronic** **Supplementary material 10** GRADE summary of findings

| **Certainty assessment** | | | | | | | **Effect** | | **Certainty** |
| --- | --- | --- | --- | --- | --- | --- | --- | --- | --- |
| **№ of studies** | **Study design** | **Risk of bias** | **Inconsistency** | **Indirectness** | **Imprecision** | **Other considerations** | **Relative (95% CI)** | **Absolute (95% CI)** |  |
| **Cognitive impairment** | | | | | | | | | |
| 5 | observational studies | serious^a^ | not serious | not serious | not serious | strong association | **RR 0.58** (0.52 to 0.65) | **1 fewer per 1,000** (from 1 fewer to 1 fewer) | ⨁⨁◯◯ Low |
| **Cognitive decline** | | | | | | | | | |
| 2 | observational studies | serious^a^ | not serious | not serious | serious^b^ | none | **RR 0.37** (0.07 to 1.85) | **0 fewer per 1,000** (from 2 fewer to 0 fewer) | ⨁◯◯◯ Very low |
| **Dementia** | | | | | | | | | |
| 10 | observational studies | serious^a^ | serious^c^ | not serious | not serious | strong association | **RR 0.73** (0.62 to 0.86) | **1 fewer per 1,000** (from 1 fewer to 1 fewer) | ⨁◯◯◯ Very low |
| **Alzheimer’s disease** | | | | | | | | | |
| 7 | observational studies | serious^a^ | serious^d^ | not serious | not serious | strong association | **RR 0.68** (0.53 to 0.87) | **1 fewer per 1,000** (from 1 fewer to 1 fewer) | ⨁◯◯◯ Very low |
| **Vascular dementia** | | | | | | | | | |
| 2 | observational studies | serious^a^ | not serious | not serious | not serious | strong association | **RR 0.48** (0.32 to 0.73) | **0 fewer per 1,000** (from 1 fewer to 0 fewer) | ⨁⨁◯◯ Low |

**CI:** confidence interval; **RR:** risk ratio

**Explanations**

a. Serious risk of bias in at least one domain

b. Wide 95% CI ranging from 0.07 to 1.85

c. I-squared of 71%

d. I-squared of 76%
